# Thermal and Calorimetric Evaluations of Some Chemically Modified Carbohydrate-Based Substrates with Phosphorus-Containing Groups

**DOI:** 10.3390/polym12030588

**Published:** 2020-03-05

**Authors:** Ananya Thomas, Paul Joseph, Khalid Moinuddin, Haijin Zhu, Svetlana Tretsiakova-McNally

**Affiliations:** 1 Institute for Sustainable Industries and Liveable Cities, Victoria University, P.O. Box 14428, Melbourne, VIC 8001, Australia; paul.joseph@vu.edu.au (P.J.); khalid.moinuddin@vu.edu.au (K.M.); 2 Institute for Frontier Materials, Deakin University, Melbourne, VIC 3125, Australia; h.zhu@deakin.edu.au; 3 Belfast School of Architecture and the Built Environment, Ulster University, Newtownabbey BT37 0QB, Northern Ireland, UK; s.tretsiakova-mcnally@ulster.ac.uk

**Keywords:** carbohydrate substrates, chemical modification, phosphorus analysis, thermogravimetric analysis, pyrolysis combustion flow calorimetry, fire-resistant coatings

## Abstract

In the present article, we report on the chemical modifications of some carbohydrate-based substrates, such as potato starch, dextran, β-cyclodextrin, agar agar and tamarind, by reacting with diethylchlorophosphate (DECP), in dispersions in dichloromethane (DCM), in the presence of triethylamine (TEA) as the base. The modified substrates, after recovery and purification, were analyzed for their chemical constitutions, thermal stabilities and calorimetric properties using a variety of analytical techniques. These included: solid-state ^31^P NMR, inductively coupled plasma-optical emission spectroscopy (ICP-OES), thermogravimetric analysis (TGA) and pyrolysis combustion flow calorimetry (PCFC). The unmodified counterparts were also subjected to the same set of analyses with a view to serving as controls. Phosphorus analyses, primarily through ICP-OES on the recovered samples, showed different degrees of incorporation. Such observations were optionally verified through solid-state ^31^P NMR spectroscopy. The thermograms of the modified substrates were noticeably different from the unmodified counterparts, both in terms of the general profiles and the amounts of char residues produced. Such observations correlated well with the relevant parameters obtained through PCFC runs. Overall, the modified systems containing phosphorus were found to be less combustible than the parent substrates, and thus can be considered as promising matrices for environmentally benign fire-resistant coatings.

## 1. Introduction

Much attention has been paid to the development of bio-degradable, carbohydrate-based materials, mainly owing to rising environmental concerns spurred by an overdependence on petro-chemically derived raw materials and polymers [1,2,3]. For example, the well-known carbohydrate starch is a natural polymer possessing a variety of advantageous properties including sustainability and bio-degradability, and its derivatives are generally non-toxic and relatively low-priced. However, in order to expand the domain of application of carbohydrate-based materials, they need to be modified, or blended, with appropriate materials with a view to enhancing their thermal stability and fire-retardant properties [2,3]. Therefore, several studies have been carried out to improve the flame-retardant efficacies of these materials, including surface modification, impregnation, and chemical modification to functionalize the hydroxyl groups of the base glucose units [4,5,6]. In this context, our long-term interest is to use phosphorus-modified systems, derived from carbohydrate-based agricultural products, as passive fire protection coatings on wood-based structural elements that are commonly used in the construction sector. The synthetic routes to phosphorylate the glucopyranose units of carbohydrate-based materials are well documented in the literature [7,8,9,10,11]. Keglevich et al. established that multiple phosphorylation reactions were possible with different propensities, and the amount of phosphorylation was found to occur predominantly in positions 6 and 3 [7,11]. These observations were supported by Blennow et al. in their comprehensive review, where steric factors were also duly considered [8]. The evidence, primarily based on the ^31^P spectrum, with regard to the exact position(s) and formation of multiple phosphorylated units was also reported [9]. In a related study, Muhammad et al. reported on the influence of pH on the degree and nature of phosphorylation of a starch substrate [10].

The usage of many halogenated compounds has been restricted due to the ecological, environmental and health issues that they often cause [2,3,12]. Hence, over the past few decades, there has been a shift in focus towards using halogen-free compounds and formulations. Besides, the flame-retardant efficiency of compounds with covalently bound phosphorus was found to be better than the usage of equally loaded halogenated compounds [5]. Consequently, phosphorus-based compounds are increasingly being used for imparting fire-retardant properties to several commercially important polymeric materials, both synthetic and natural. In addition, phosphorus-containing compounds, when used as additives, were proven to facilitate the thermal degradation of cellulose, and favored the formation of solid products instead of releasing volatile gases [1,13]. Furthermore, phosphorus-based compounds seem to work better for cellulosic materials since they contain a significant number of hydroxyl groups, some of which can be functionalized, thereby increasing their char-forming tendency and hence their fire-retardant properties [1,8,14,15].

With this mind, we have endeavored to chemically modify some common carbohydrate-based substrates, such as potato starch, dextran, β-cyclodextrin, agar agar and tamarind, where all the substrates not only widely differed in terms of their molecular weights but also in terms of their chemical structures. We chose to carry out a simple condensation reaction, in the presence of a base (TEA), with a view to covalently binding the phosphorus group of interest (i.e., phosphate functional group) for obvious reasons. These mainly include: low levels of loadings might suffice; once covalently bound, the fire-retardant moieties are less likely to leach out from the base substrates; and the favored spatial dispositions of pendent modifying groups make them more amenable for intra-chain condensation reactions and/or neighboring group participation in bringing about char formation reactions. The recovered polymers were subjected to a variety of characterization techniques, primarily to gain insights into their chemical natures, and thermal and combustion properties. Furthermore, we have attempted to correlate some of the relevant parameters obtained through thermogravimetric analysis (TGA) runs and pyrolysis combustion flow calorimetry (PCFC) measurements.

## 2. Materials and Methods 

### 2.1. Materials

**Figure 1:**
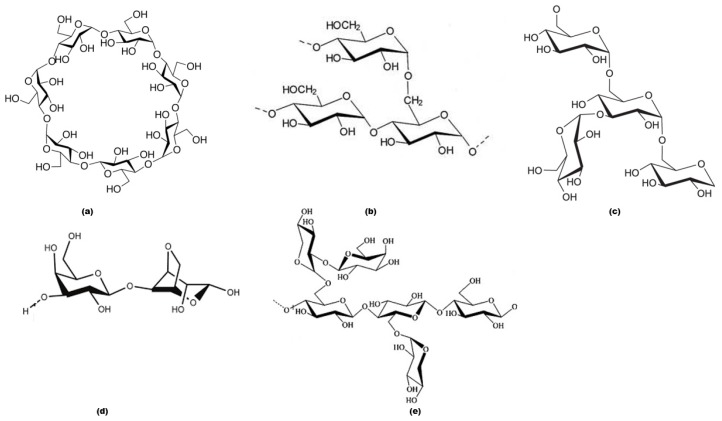
Chemical structures of the substrates: (**a**) β-cyclodextrin, (**b**) potato starch, (**c**) dextran, (**d**) agar agar, (**e**) tamarind.

The carbohydrate-based materials selected for the current study included potato starch, dextran, β-cyclodextrin and agar agar, which were obtained from Aldrich Chemical Company, Melbourne, Victoria, Australia, whereas tamarind kernel powder was sourced locally (Figure 1). The reagents, solvents and other chemicals used for the preparative procedures, including diethylchlorophosphate (DECP), triethylamine (TEA), dichloromethane (DCM) and concentrated nitric acid (AR grade), were also purchased from Aldrich Chemical Company. All the substrates, reagents and solvents were used as received, unless specified. Generally, DECP, TEA and DCM were dried and stored over molecular sieves (4 Å) prior to use.

### 2.2. Methods

#### 2.2.1. Synthetic Procedures

##### Chemical Modification of the Base Substrates

A typical procedure on a ca. 2–3 g scale was as follows: approximately 2.5 g of each material (potato starch, or dextran, or β-cyclodextrin, or agar agar, or tamarind), as the case may be, was taken in a 250 cm^3^ conical flask, and about 40 cm^3^ of dry dichloromethane and ca. 5 cm^3^ of TEA were added to the substrate and the flask was stoppered. To this mixture, 2.2 cm^3^ of dry diethylchlorophosphate was introduced, over a period of 30 min, and the contents were stirred at room temperature for about 24 h. After the required reaction period, the content of the conical flask was filtered under a vacuum, through a qualitative filter paper, in a Buckner funnel. The solid residue that was collected onto the filter paper was thoroughly washed with deionized water in order to ensure that all unreacted DECP was washed out (at least six times with 50 cm^3^ of deionized water). The product thus obtained was dried in a hot air oven (ca. 70 °C) for at least 72 h (see Table 1 for details).

#### 2.2.2. Characterization Techniques

The primary aim of the characterization was to calculate the extent of incorporation of phosphorus-containing groups (primarily through inductively coupled plasma-optical emission spectroscopy (ICP-OES)), and also to gauge the thermal and calorimetric properties of the modified materials. For the latter purpose, thermogravimetric analysis (TGA) and pyrolysis combustion flow calorimetry (PCFC) were employed. The ^31^P solid-state NMR spectrum of the modified starch provided conclusive proof of phosphorus functionalization of the base matrix. The unmodified counterparts were also subjected to the same set of analyses for the purpose of comparison.

##### Spectroscopic Analyses (Solid-State NMR)

The solid-state NMR (^31^P with cross-polarization/magic angle spinning: CP-MAS mode) was obtained by employing a 500 MHz Bruker machine at ambient probe conditions, typically at 10 kHz rotor speed, and the signals were calibrated against phosphoric acid as the external calibrant. The raw data were then processed by using proprietary software from the manufacturer, Bruker Pty Ltd., Melbourne, Victoria, Australia (TopSpin 4.0.6). 

##### Inductively Coupled Plasma-Optical Emission Spectroscopy (ICP-OES)

The samples were first accurately weighed (ca. 10–15 mg, in triplicate), and then were digested by boiling with 5 cm^3^ of analytical grade concentrated HNO_3_ in 50 cm^3^ beakers. Following the digestion of the unmodified samples and modified versions of dextran, β-cyclodextrin, potato starch, agar agar and tamarind (as the case may be), the resulting solutions were transferred quantitatively into 25 cm^3^ volumetric flasks and the volumes were adjusted to the marks with deionized water. In the case of an incomplete digestion, as inferred from the presence of solid residues, the contents were first filtered using a filter before making up to the required volumes. The prepared solutions were transferred to 15 cm^3^ tubes before being injected into an ICP-OES instrument, Shimadzu ICPE-9000 (Shimadzu Scientific Instruments, Melbourne, Victoria, Australia). Each of the samples was repeated three times, so as to attain a more accurate value of the phosphorus content in each sample. The quantitative assessments of the phosphorus contents in the samples (see in Table 2) were obtained through constructing a Beer–Lambert plot.

##### Thermogravimetric Analysis (TGA)

In the present study, TGA runs were performed on samples (ca. 5–10 mg) under an atmosphere of nitrogen, in the temperature range from 30 to 800 °C, at a heating rate of 60 °C/min, using a Mettler-Toledo instrument. The relatively higher heating rate (i.e., 60 °C/min) was specifically chosen for the TGA experiments with a view to match it with the corresponding heating rate that was employed in the PCFC runs (i.e., 1 °C/s) (Figures 3–7). The reproducibility of the thermogram’s rates was periodically checked by performing duplicate/triplicate runs.

##### Pyrolysis Combustion Flow Calorimetry (PCFC)

This is a small-scale experimental technique used for evaluating the general flammability behaviors of materials. PCFC works on the principle of oxygen consumption calorimetry. It is also known as micro-scale combustion calorimetry, in which a very small sample size (ca. 25–40 mg) provides a wide range of combustion/flammability-related data. This process is carried out in an inert gas stream of nitrogen, or in a mixture of oxygen and nitrogen, with high temperatures to facilitate oxidation of the volatile products of pyrolysis. The useful parameters that were obtained through PCFC include heat release rates, total heat released, mass of the residue left, effective heats of combustion and the heat release capacity [16]. In the present study, we employed an FAA Micro Calorimeter to obtain the plots of heat release rates (HRRs) versus the temperatures (Figures 8–12). The samples were pyrolyzed in an inert atmosphere (i.e., in nitrogen), and pyrolytic gases were sent to a combustor at 900 °C in the presence of oxygen in excess. The mass of the samples was adjusted based on their respective oxygen consumption values (ca. 7–13%).

## 3. Results and Discussion

**Figure 2:**
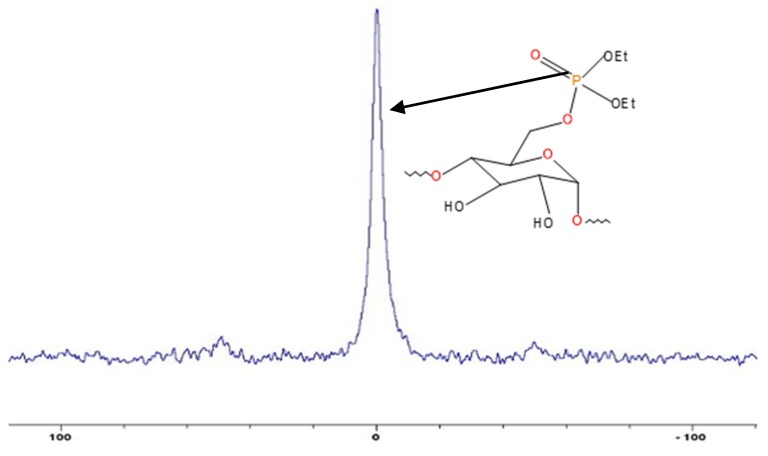
^31^P-NMR spectrum of potato starch modified with phosphate groups (the abscissa denotes the chemical shift values, δ, in ppm, and the ordinate corresponds to the signal intensity in arbitrary units).

The chemical incorporation of the phosphorus-containing groups (i.e., phosphate) was clearly evident from the solid-state NMR spectrum in the case of potato starch (see Figure 2). In the case of all the other substrates (i.e., β-cyclodextrin, dextran, agar agar and tamarind), such a modification reaction was primarily inferred from ICP-OES measurements. Table 1 summarizes the details of the preparative procedures including the recovered yields and the corresponding phosphorus loadings (as wt. % obtained through ICP-OES measurements). Phosphorus estimations of unmodified substrates were also carried as blank runs, which showed no detectable levels according to ICP-OES measurements. It should be noted here that the coupling reactions between the hydroxyls (OH) in the substrate and the diethylchlorophosphate (DECP) were attempted in the presence of a base (TEA). Here, the base was expected to act as an acid scavenger, aiding in the removal of the product of condensation (HCl) between the hydroxyl groups (presumably in positions 3 and 6 of the glucopyranose rings) and the reagent, DECP [7,8,9,10,11]. 

**Figure 3:**
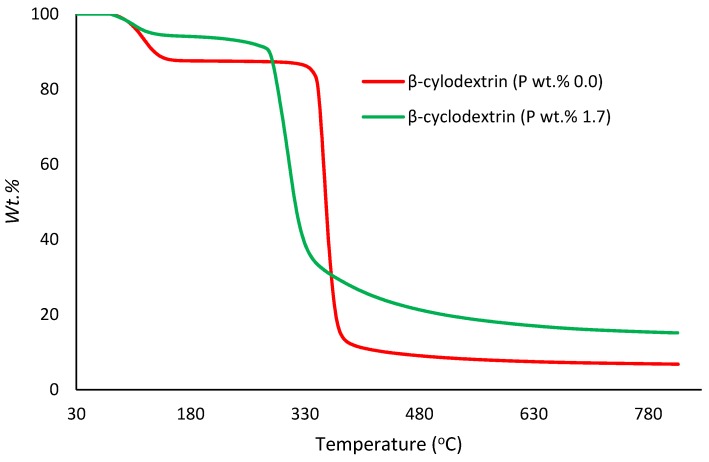
Thermograms of modified and unmodified versions of β-cyclodextrin at 60 °C/min.

**Figure 4:**
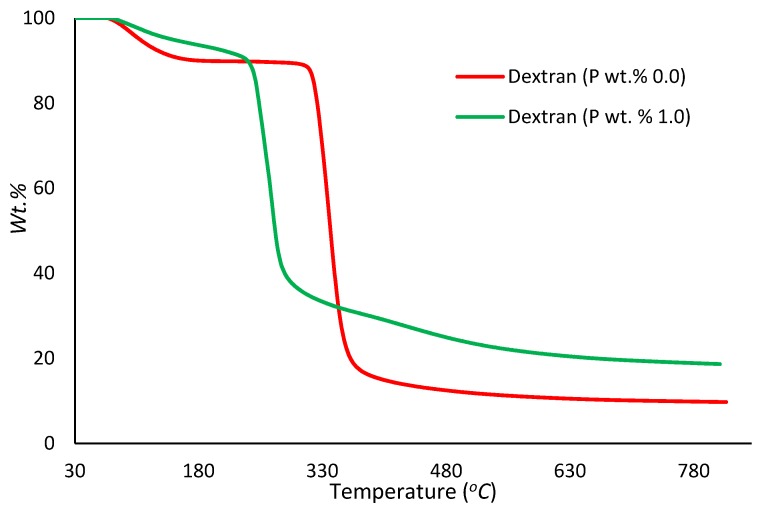
Thermograms of modified and unmodified versions of dextran at 60 °C/min.

**Figure 5:**
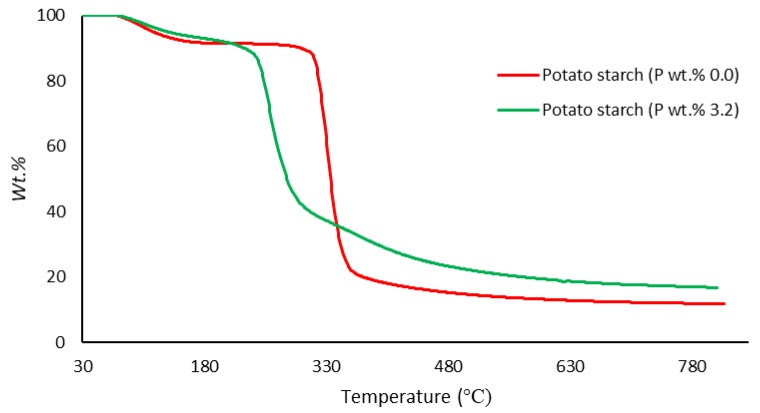
Thermograms of modified and unmodified versions of potato starch at 60 °C/min.

**Figure 6:**
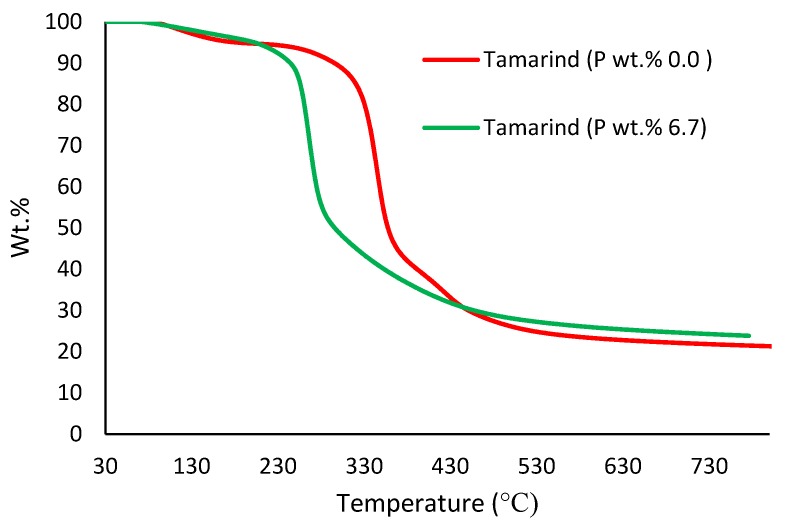
Thermograms of modified and unmodified versions of tamarind at 60 °C/min.

**Figure 7:**
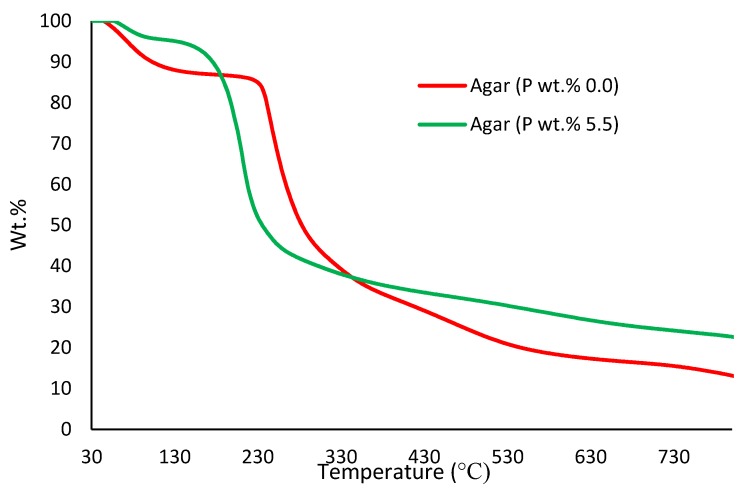
Thermograms of modified and unmodified versions of agar agar at 60 °C/min.

Figure 3, Figure 4, Figure 5, Figure 6 and Figure 7 show the thermograms of β-cyclodextrin, dextran, potato starch, agar agar and tamarind. It can be clearly seen that the general profiles, induction temperatures and the amounts of the char residue obtained at 800 °C differed markedly with the modified versions as compared with the unmodified material in all cases (see Table 3 for details).

**Figure 8:**
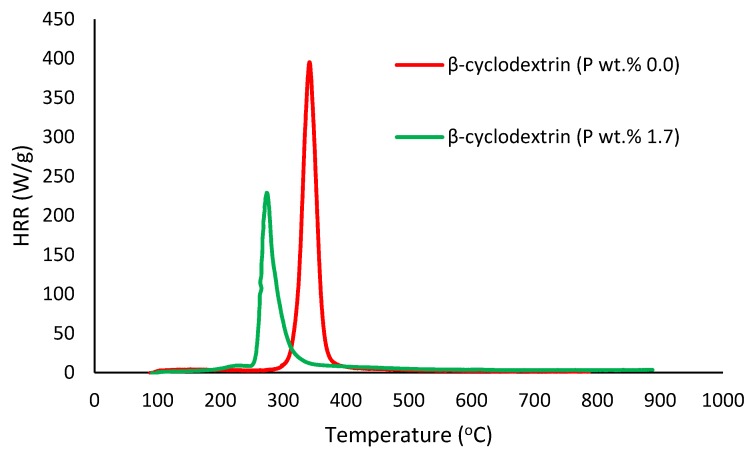
Plot of the heat release rate (HRR) (W/g) versus temperature (°C) for modified and unmodified versions of β-cyclodextrin.

**Figure 9:**
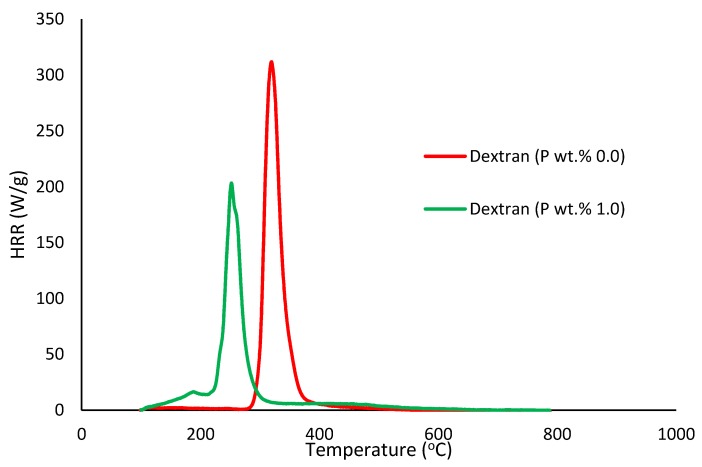
Plot of heat release rate (HRR) (W/g) versus temperature (°C) for modified and unmodified versions of dextran.

**Figure 10:**
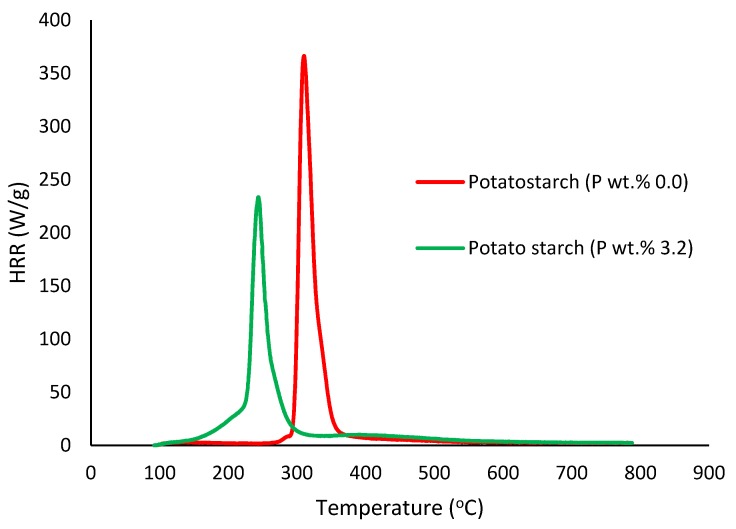
Plot of heat release rate (HRR) (W/g) versus temperature (°C) for modified and unmodified versions of potato starch.

**Figure 11:**
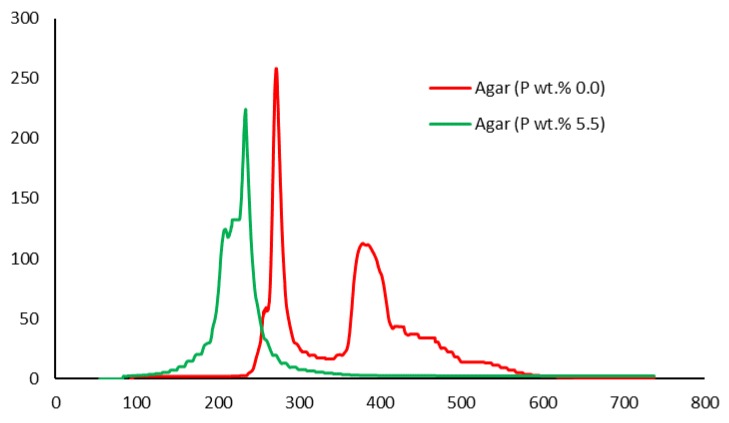
Plot of heat release rate (HRR) (W/g) versus temperature (°C) for modified and unmodified versions of agar.

**Figure 12:**
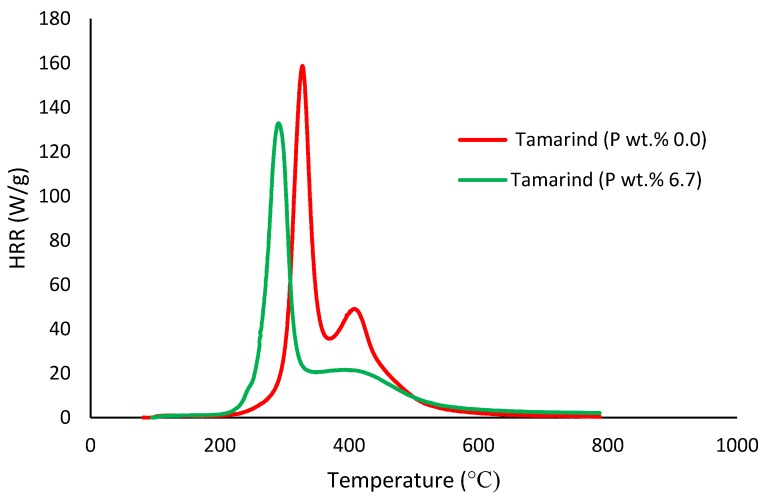
Plot of heat release rate (HRR) (W/g) versus temperature (°C) for modified and unmodified versions of tamarind.

It is quite evident that the incorporation of phosphorus-bearing moieties (i.e., phosphate groups) clearly changes the degradation pathways of all the substrates. These effects are reflected in the somewhat altered values for the induction temperature, and in the lower temperature ranges for the primary degradation steps coupled to a slower rate of degradation, and in finally producing higher char yields for the modified systems as compared to the unmodified matrices. It is also interesting to note that the temperature at 50 wt. % showed the minimum value in the case of phosphorus-modified samples except in the case of potato starch. In addition, the temperature corresponding to the peak heat release rates (pHRRs) showed the same trend in all cases. Overall, the thermal and calorimetric behaviors of the modified materials are indicative of their lower combustibility attributes in comparison with the unmodified counterparts. It should be noted here that the degree of alteration of the thermal degradation characteristics is in tune with the phosphorus loadings. This inference could be corroborated with the relevant values obtained from the PCFC curves (see Figure 8, Figure 9, Figure 10, Figure 11 and Figure 12 and Table 3). 

Generally, phosphorus-modified samples very clearly exhibited earlier combustion exothermic peaks on thermograms, as compared to the corresponding unmodified materials, presumably owing to the thermal cracking of the modifying phosphate groups [17]. Concomitantly, acidic species derived from the phosphorus functionalities could trigger dehydration reactions of the glucose units in the substrates, thus promoting char production. The latter effect is reflected in the enhanced values of char residue yields, as recorded by the TGA and PCFC measurements (see Table 2 and Table 3). The enhancements in char yields often result in carbon-rich residues, thus resulting in decreases in the values of the relevant combustion parameters, such as pHRR, THR and HRC, obtained through the PCFC measurements, including the calculated values of the effective heats of combustion.

Apparent values of heats of combustion (*h_c_*) of solid fuels can be calculated from the THR values normalized to the mass fractions of the material that underwent combustion, where the required denominator can be obtained from the initial mass of the material and amount of char residue obtained [16].

$$h_{c}=\frac{THR}{1-Y_{p}}$$

Here, *THR* is the specific heat release of the sample, i.e., the area under the corresponding HRR curve (kJ/g); $Y_{p}$ is the pyrolysis residue (g/g).

The values so obtained are given in Table 4. The relatively lower values for the apparent heats of combustion, which in turn seemed to depend on the extent of phosphorus loading, can be attributed to the changes in the amounts and/or nature of the pyrolysis gases generated by the first stage of the experiment.

**Figure 13:**
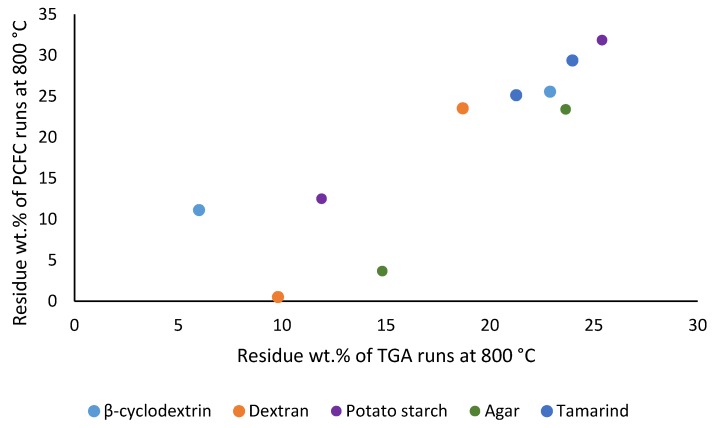
Correlation of char residues (wt. %) from TGA experiments and PCFC runs of the substrates.

**Figure 14:**
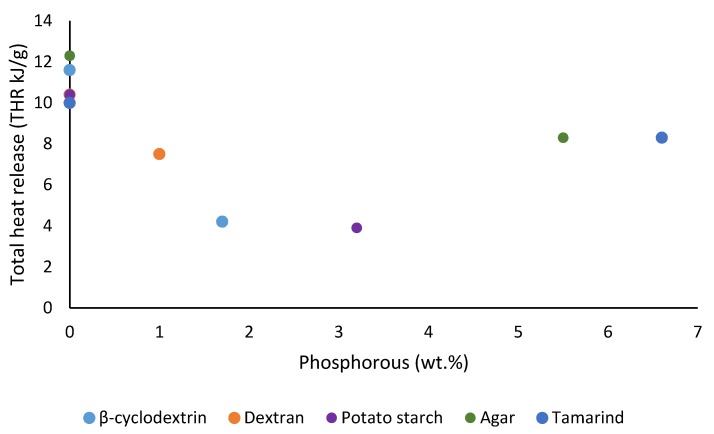
Plot of total heat release (THR) versus phosphorus content (wt. %) of the substrates.

**Figure 15:**
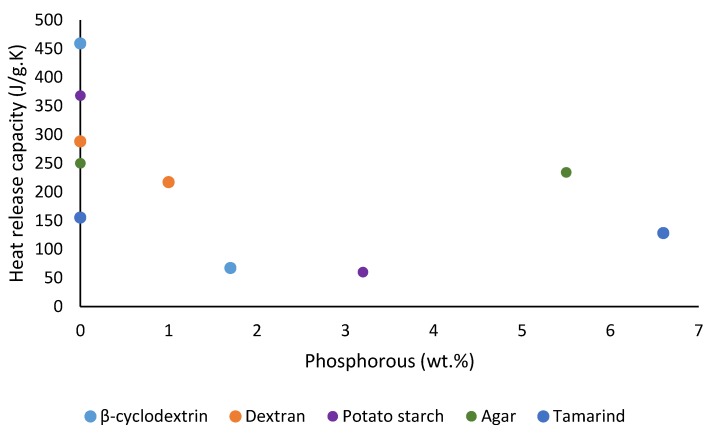
Plot of the heat release capacity versus phosphorus content (wt. %) of the substrates.

**Figure 16:**
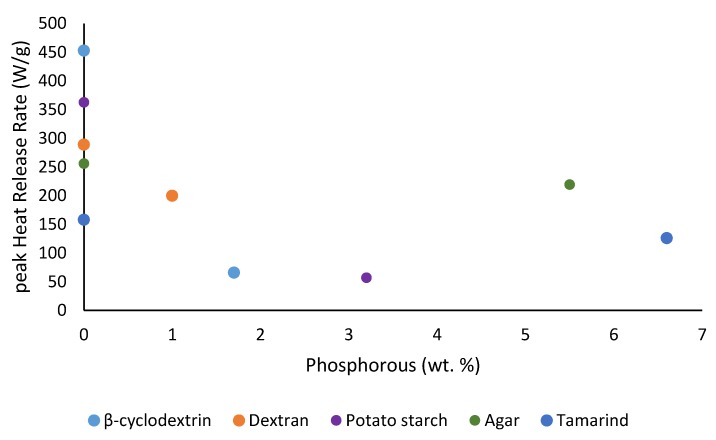
Plot of peak heat release rates (pHRR) versus phosphorus content (wt. %) of the substrates.

**Figure 17:**
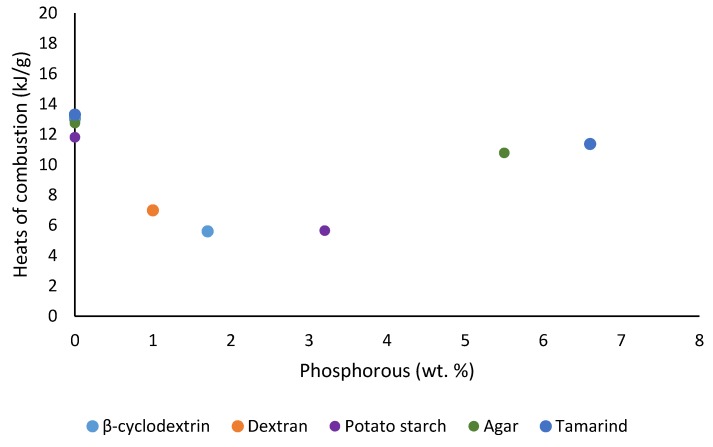
Plot of heats of combustion (*h_c_*) versus phosphorus content (wt. %).

We also noticed some degree of correlation of the results from TGA and PCFC in terms of the amounts of char residues obtained for all the substrates (see Figure 13), where the samples underwent degradation reaction(s) in nitrogen. The degradative processes in a TGA experiment can be thought of as the fuel production stage (hence the amount of combustible volatiles) in the case of solid fuels. Therefore, this should be considered as the first stage in a PCFC experiment, where the material is pyrolyzed in a nitrogen atmosphere. It can be seen here that there is a noticeable relationship between the flame retardancy effect of the phosphorus-containing groups and their loadings (Figure 14, Figure 15, Figure 16 and Figure 17). As mentioned earlier, phosphorus modification of the base substrates results in altered degradative pathways, thus changing the nature and composition of the pyrolysis gases. This is in agreement with literature precedents, especially in the case of some commercially important thermoplastics, where acceptable levels of fire retardation are observed with phosphorus loadings achieved through a reactive strategy between 2.0 and 5.0 wt. %, whereas any higher loadings often result in diminished returns [18,19,20]. 

## 4. Conclusions

In the present work, we have successfully modified five different types of polymeric and oligomeric carbohydrates (potato starch, dextran, β-cyclodextrin, agar agar and tamarind), by covalently binding phosphate groups through the reaction of diethylchlorophosphate (DECP) with the substrates in dispersions in dichloromethane (DCM), in the presence of trimethylamine (TEA). The chemical constitutions of the modified substrates were, optionally, inferred from solid-state NMR spectroscopy, and the extents of the phosphorus loadings were determined through inductively coupled plasma-optical emission spectroscopy (ICP-OES). The thermal and calorimetric properties of the modified materials were primarily assessed through thermogravimetric analysis (TGA) and pyrolysis combustion flow calorimetric (PCFC) measurements. The experimental parameters obtained for the modified versions were also compared with the corresponding empirical values for the unmodified counterparts. 

Phosphorus incorporation, through covalent linkages to the hydroxyl groups of the carbohydrate substrates, has clearly influenced the thermal degradation characteristics and combustion attributes of the base materials. Generally, the modified materials produced larger amounts of char residues, thus resulting in lesser amounts of combustible volatiles. The latter aspect was clearly evident from the PCFC runs. These effects can be attributed to the presence of covalently bound phosphate groups in the modified systems. Furthermore, noticeable degrees of flame retardancy effect in the modifying groups were obtained through nominal phosphorus loading (ca. 2–4 wt. %) achieved through a reactive strategy. Thus, the present work opens up the tangible prospect of formulating fire-resistant water-borne coatings, incorporating modified carbohydrates, both natural and synthetic, as polymeric materials. Following on from this, the most promising systems can be scaled up with a view to exploring the commercial viability of such products. 

## Figures and Tables

**Table 1:** Details of the chemical modification reactions.

Sl. No.	Substrate	Sample Weight (g)	DECP (g)	DCM (cm^3^)	TEA (cm^3^)	Recovered Yield (wt %)	P (wt %)
1	β-cyclodextrin	2.5	2.63	40	5	49.90	1.7
2	Dextran	2.4	2.63	40	5	79.15	1.0
3	Potato starch	2.3	2.63	40	5	55.17	3.2
4	Agar agar	2.7	2.63	40	5	66.45	5.55
5	Tamarind	2.8	2.63	40	5	68.23	6.68

**Table 2:** Details from thermogravimetric analysis (TGA).

Sample	P (wt %)	Induction Temperature (°C)	Temperature at 50 wt % (°C)	Residue at 400 °C (wt %)	Final Residue at 800 °C (wt %)
β-cyclodextrin	0.0	94.0	356	11.4	6.00
β-cyclodextrin	1.7	96.0	334	39.7	22.9
Dextran	0.0	85.0	340	15.2	9.80
Dextran	1.0	93.0	273	29.3	18.7
Potato Starch	0.0	93.0	335	18.3	11.9
Potato Starch	3.2	129	366	45.6	25.4
Agar agar	0.0	77.0	333	36.7	14.8
Agar agar	5.5	90.0	280	36.9	23.7
Tamarind	0.0	86.0	357	38.5	21.3
Tamarind	6.6	105	338	39.6	24.0

**Table 3:** Pyrolysis combustion flow calorimetry (PCFC) data for modified and unmodified versions of base substrates (average values of triplicate runs).

Sample	P (wt %)	Temp to pHRR (°C)	pHRR (W/g)	THR (kJ/g)	Heat Release Capacity (J/g K)	Char Yield (wt %)
β-cyclodextrin	0.0	342	452.7	11.6	459	11.11
β-cyclodextrin	1.7	290	66.02	4.20	67.0	25.54
Dextran	0.0	319	289.0	10.4	288	<1.0
Dextran	1.0	252	199.9	7.50	217	23.52
Potato Starch	0.0	310	362.8	10.4	368	12.50
Potato Starch	3.2	260	57.18	3.90	60.0	31.85
Agar agar	0.0	272	256.0	12.3	250	3.680
Agar agar	5.5	234	219.4	8.30	234	23.40
Tamarind	0.0	326	158.0	10.0	155	25.12
Tamarind	6.6	291	126.0	8.10	128	29.36

**Table 4:** Heats of combustion values.

Sample	P (wt %)	Pyrolysis Residue (g/g)	*h_c_* (kJ/g)
β-cyclodextrin	0.0	0.11	13.03
β-cyclodextrin	1.7	0.25	5.600
Dextran	0.0	— *	—
Dextran	1.0	0.27	6.980
Potato Starch	0.0	0.23	11.81
Potato Starch	3.2	0.31	5.650
Agar agar	0.0	0.036	12.75
Agar agar	5.5	0.23	10.78
Tamarind	0.0	0.25	13.30
Tamarind	6.6	0.27	11.36

* Pyrolysis residue could not be measured accurately as the sample expanded considerably and spilled over the pan upon the degradation/combustion.
